# A case of herbicide-induced acute fibrinous and organizing pneumonia?

**DOI:** 10.1186/s12890-017-0547-7

**Published:** 2017-12-13

**Authors:** Shengsong Chen, Hong Zhou, Lingling Yu, Bo Tong, Zuke Xiao, Sisi Fan

**Affiliations:** 1 Department of Respiratory and Critical Care Medicine, Jiangxi Provincial People’s Hospital, No.92 Aiguo Road, Nanchang, 330006 China; 2 Department of Cardiology, the Second Affiliated Hospital of Nanchang University, No.1 Minde Road, Nanchang, 330006 China; 3 Department of Pathology, Jiangxi Provincial People’s Hospital, No.92 Aiguo Road, Nanchang, 330006 China

**Keywords:** AFOP, Pathology, CT

## Abstract

**Background:**

To improve the understanding of acute fibrinous and organizing pneumonia (AFOP), we present one case of AFOP proven by percutaneous lung biopsy along with clinical features, chest imaging and pathology.

**Case presentation:**

A 50-year-old man was admitted to our department after he was given empiric therapy for community-acquired pneumonia (CAP). The clinical symptoms of the patient were dry cough, chills, night sweats and high fevers. Chest computed tomography (CT) scan showed a high-density shadow in the right lung lobe, similar to lobular pneumonia. The patient was preliminarily diagnosed with community-acquired pneumonia; however, antibacterial treatment was ineffective. To confirm the diagnosis, we performed bronchoscopy and percutaneous lung biopsy; pathology was consistent with AFOP. After he was treated with glucocorticoids, the patient’s symptoms were relieved, and the shadow seen on imaging dissipated during the follow-up period.

**Conclusions:**

AFOP is a rare histopathological diagnosis that can be easily misdiagnosed. Clinicians need to consider the possibility of AFOP in the case of invalid antibacterial therapy.

## Background

Ever since the concept of AFOP was proposed by Beasley in a pathologic study of 17 patients with acute/subacute lung injury in 2002 [1], more and more cases have been published across all age groups. However, AFOP has not been included in the clinical category of idiopathic interstitial pneumonia (IIP) but has been referred to as a rare pathological type in recent years [2]. Interestingly, AFOP symptoms are not typical; patients commonly present with dyspnoea, cough, fever, etc., which makes it more difficult to diagnose compared to other diseases [1, 3, 4]. In addition, the treatment of AFOP is controversial; both glucocorticoids and immunosuppressive agents have been shown to be effective [1, 3, 4]. To improve the understanding of AFOP, we herein present one case of a patient with AFOP proven by pathology, whose clinical symptoms were significantly relieved by glucocorticoid treatment.

## Case presentation

### General information

A 50-year-old male farmer, a declared nonsmoker, with history of contact with glyphosate (a kind of herbicide) 2 days prior to symptom onset, was admitted to our department with a 20-day history of dry cough, chills, night sweats and high fevers on October 6. He was administered empiric therapy for community-acquired pneumonia (CAP) with piperacillin-tazobactam and treatment was invalid at a local hospital.

### Physical examination

On his admission, vital signs were as follows: temperature, 40 °C, oxygen saturation on room air, 95%. Chest auscultation revealed breath sounds with fine crackles and wheezes increased in the right lung; no other findings were remarkable.

### Auxiliary examination

**Fig. 1:**
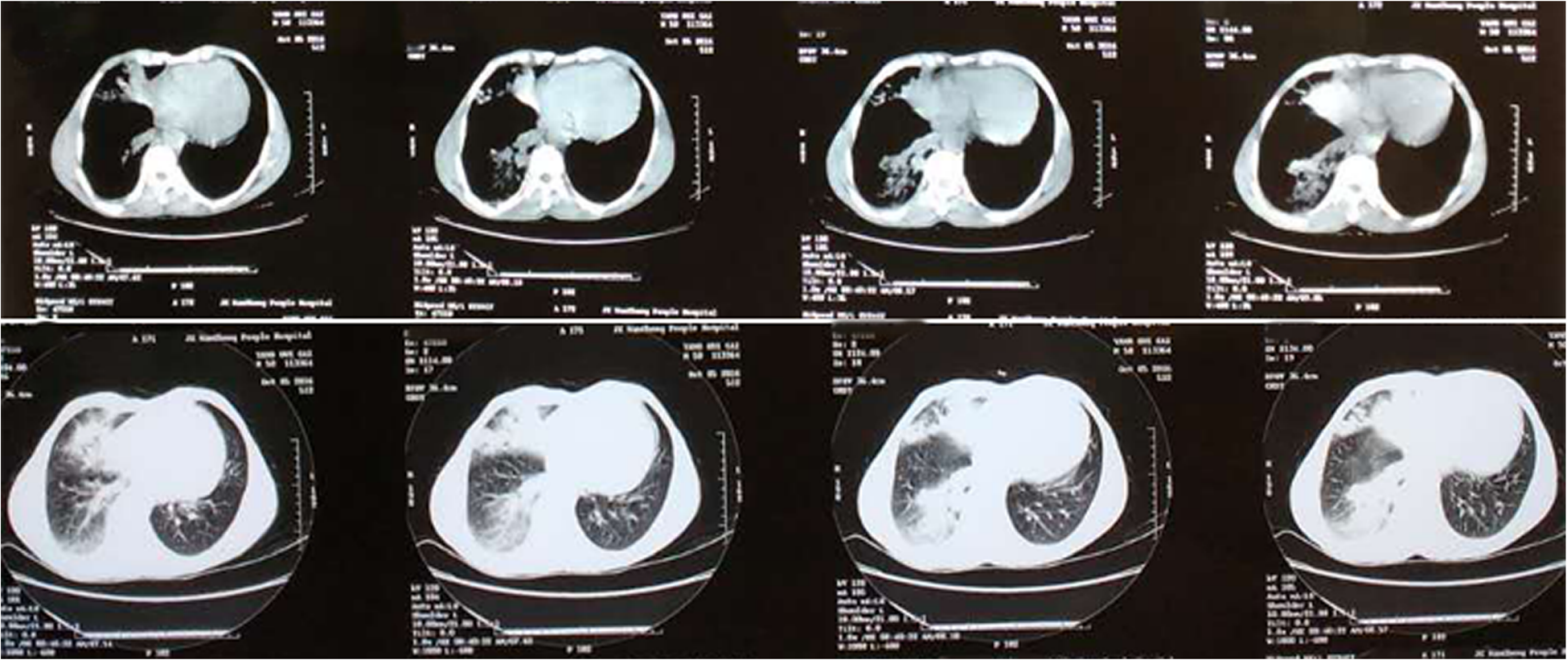
The local hospital chest CT (Oct. 01) showed patchy opacities and a spot-like high density shadow in the right basement of the lower lobe and the right middle lobe

The local hospital chest CT (Fig. 1) showed patchy opacities and a spot-like high density shadow in the right basement of the lower lobe and the right middle lobe. The initial bloodwork was as follows: WBC19.2 × 10^9^/L, N85%, L8%. The procalcitonin level was 1.22 ng/ml, ESR was 72 mm/h. The other laboratory tests all were negative, including rapid antigen tests for influenza and HIV; liver, kidney and coagulation function tests; arterial blood gas analysis; autoimmune and tumour biomarkers; G-test and lipopolysaccharide; blood, bone marrow and sputum culture; and detection of herbicide toxicity.

Preliminary diagnosis: community-acquired pneumonia.

## Treatment course

**Fig. 2:**
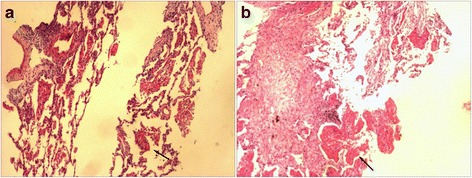
Haematoxylin and eosin (H&E) stain, massive cellulose (arrow) exudate with organization in the alveolar spaces, alveolar septum widened with oedema and lymphocytes and sparse eosinophilic infiltration. No necrosis, bleeding or neutrophil infiltration could be seen. **a** bronchoscopy (original magnification × 10). **b** ultrasound-guided percutaneous needle lung biopsy (original magnification × 20)

**Fig. 3:**
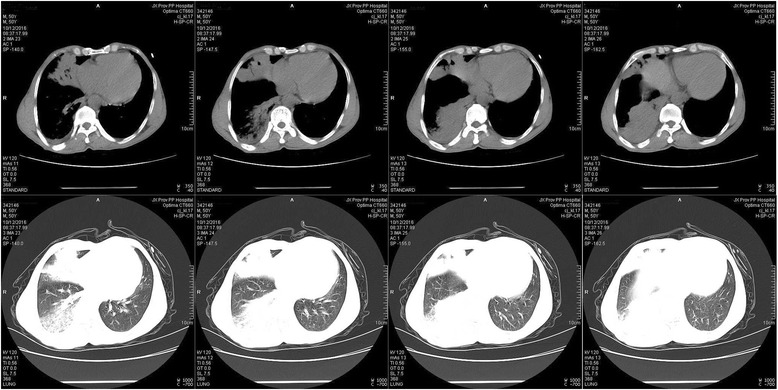
The first Chest CT (Oct. 12) showed a spot-like, high-density shadow enlarged as before

**Fig. 4:**
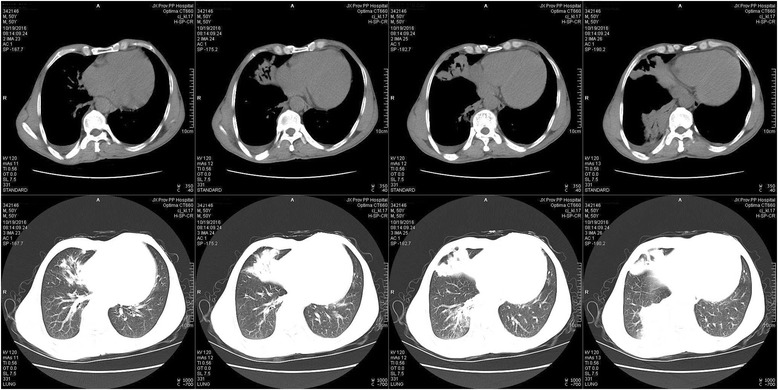
The second Chest CT (Oct. 19) showed the shadow had absorbed

**Fig. 5:**
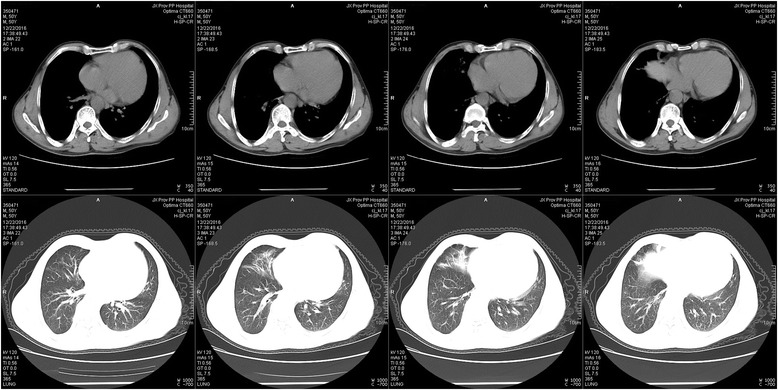
The third Chest CT (Dec. 22) showed the shadow had basically resolved, with a residual fibre cable

On admission, considering drug-resistant pneumonia, the patient was treated empirically with levofloxacin plus imipenem/cilastatin, imipenem/cilastatin plus vancomycin and anti-tuberculosis treatment in succession; however, symptoms were without remission. Bronchoscopy was conducted, and staining for acid-fast bacillus and fungus was negative in the bronchoalveolar lavage fluid (BALF), but the pathology study showed (Fig. 2a) massive cellulose exudate under the microscope. Chest CT scan (Fig. 3) showed no improvement, same as before. In view of the situation of invalid antibacterial treatment and exclusion of other infection diseases, the patient was administered methylprednisolone 80 mg daily; the patient’s fever subsided and symptoms improved significantly. To confirm the diagnosis, we conducted an ultrasound-guided percutaneous needle lung biopsy; pathology revealed (Fig. 2b) massive cellulose exudate with organization in the alveolar cavity, alveolar septum widened with oedema and lymphocytes and sparse eosinophilic infiltration. No necrosis, bleeding or neutrophil infiltration could be seen. Above all, we considered a diagnosis of AFOP; the patient was continued on methylprednisolone 80 mg daily without obvious discomfort. After 5 days, the dose was changed to 40 mg daily. Repeat chest CT scan (Fig. 4) revealed the opacity had reduced in size. After another 3 days, the patient was switched to prednisone 40 mg orally, with a reduction of 5 mg weekly after discharge from our hospital. During the follow-up period, repeat chest CT scan (Fig. 5) showed resolution was achieved and the patient remained asymptomatic.

Final diagnosis: acute fibrinous and organizing pneumonia (AFOP).

## Discussion and conclusions

A literature review revealed that fewer than 120 cases have been published; whether AFOP can be treated as an independent disease or whether it was a distinct pattern of acute lung injury (ALI) remained to be elucidated.

To the best of our knowledge, AFOP diagnosis depends mainly on pathology. The differential diagnosis includes as follows, organizing pneumonia (OP), eosinophil pneumonia (EP), and diffuse alveolar damage (DAD) [5, 6]. Interestingly, AFOP has distinctive histopathology, characterized by massive cellulose exudate with organization in the alveolar spaces, rather than the fibrous tissue and fibroblast proliferation seen in OP; the numerous eosinophils, macrophage infiltration and eosinophil abscesses formed in EP; or the hyaline membranes seen in DAD [5, 6]. Some scholars have suggested AFOP might be the late pathologic changes of ALI, associated with alveolar wall capillary damage and bleeding [7].

AFOP could be idiopathic or could also be associated with other diseases, lung transplantation [8], connective tissue disease [9, 10], infection [11, 12], drug reactions [13], etc. For this case, the patient was a general farmer with a history of contact with an herbicide prior to symptom onset. As a result, could we boldly speculate the pesticide was also an independent risk factor? Glyphosate is similar to paraquat, which is widely used in China’s rural areas. As a low-toxicity herbicide, only a few poisoning cases have been reported [14]. Although glyphosate may not cause pulmonary fibrosis as severe as paraquat, it still might lead to lung injury, including multi-organ damage. Of course, our speculation was done to only improve the understanding of AFOP; the relevant mechanisms need to be further clarified via experiments.

In conclusion, AFOP, which is referred to as a rare histopathological type, can easily be misdiagnosed. Clinicians need to take into consideration the possibility of AFOP in the case of invalid antibacterial therapy. This case was not novel but of significant clinical importance and very instructive.

## Abbreviations

AFOP: Acute fibrinous and organizing pneumonia
ALI: Acute lung injury
CAP: Community-acquired pneumonia
DAD: Diffuse alveolar damage
EP: Eosinophil pneumonia
H&E: Haematoxylin and eosin
OP: Organizing pneumonia
